# Are 30 minutes of rest between two incremental shuttle walking tests enough for cardiovascular variables and perceived exertion to return to baseline values?

**DOI:** 10.1590/bjpt-rbf.2014.0078

**Published:** 2015-03-13

**Authors:** Laís R. G. Ribeiro, Rafael B. Mesquita, Laís S. Vidotto, Myriam F. Merli, Débora R. Carvalho, Larissa A. de Castro, Vanessa S. Probst

**Affiliations:** 1 Centro de Pesquisa em Ciências da Saúde (CPCS), Universidade Norte do Paraná (UNOPAR), Londrina, PR, Brazil; 2 Curso de Fisioterapia, Universidade de Cuiabá (UNIC), Cuiabá, MT, Brazil; 3 Laboratório de Pesquisa em Fisioterapia Pulmonar (LFIP), Departamento de Fisioterapia, Universidade Estadual de Londrina (UEL), Londrina, PR, Brazil

**Keywords:** rehabilitation, exercise test, arterial pressure, heart rate, dyspnea, fatigue

## Abstract

**Objective:**

To verify whether 30 minutes of rest between two incremental shuttle walking tests (ISWT) are enough for cardiovascular variables and perceived exertion to return to baseline values in healthy subjects in a broad age range.

**Method:**

The maximal exercise capacity of 334 apparently healthy subjects (age ≥18) was evaluated using the ISWT. The test was performed twice with 30 minutes of rest in between. Heart rate (HR), arterial blood pressure (ABP), dyspnea, and leg fatigue were evaluated before and after each test. Subjects were allocated to 6 groups according to their age: G1: 18-29 years; G2: 30-39 years; G3: 40-49 years; G4: 50-59 years; G5: 60-69 years and G6: ≥70 years.

**Results:**

All groups had a good performance in the ISWT (median >90% of the predicted distance). The initial HR (HRi) of the second ISWT was higher than the first ISWT in the total sample (p<0.0001), as well as in all groups (p<0.0001). No difference was observed in the behavior of ABP (systolic and diastolic) and dyspnea between the two tests, but this difference occurred for leg fatigue (greater before the second ISWT) in G1 (p<0.05). Most subjects (58%) performed better in the second test.

**Conclusion:**

30 minutes of rest between two ISWTs are not enough for all cardiovascular variables and perceived exertion to return to baseline values. However, this period appears to be sufficient for blood pressure and performance to recover in most subjects.

## Introduction

The evaluation of maximal exercise capacity has been considered an important assessment in different situations and populations over the last years, aiming to describe a healthy population profile and verify impairment in diseases such as congestive cardiac failure^1^, chronic obstructive pulmonary disease (COPD)^2^, and interstitial pulmonary disease^3^.

The cardiopulmonary exercise test (CPET) is considered the gold standard to evaluate maximal exercise capacity, however its use is limited since it is time-consuming and requires expensive equipment and specially trained staff^4^. Alternatively, field tests such as the Incremental Shuttle Walking Test (ISWT) have been largely used due to their applicability and validity^5^.

The ISWT is a simple and low-cost test which consists of a 10-meter route and 12 increasing levels of speed determined by audio signals^5^. The distance covered is the main variable used for analysis, indicating the maximal exercise capacity^5^. However, this single variable does not reflect the body's response to maximal exercise. Therefore, it is common to verify physiological parameters, such as heart rate (HR), arterial blood pressure (ABP), peripheral oxyhemoglobin saturation (SpO_2_), and symptoms of perceived exertion (dyspnea and fatigue) before, during, and after the test. The goal is to ensure maximal exercise and the safety of the subjects and to keep the physiological parameters within normal range values in order to avoid complications. In addition, it is possible to use some of these measured variables to calculate other parameters, such as the heart rate reserve (HHR), the double product (DP), and the chronotropic incompetence (CI), which add important information regarding the body's response to exercise^6^.

Two ISWTs have often been performed with an interval of 20 to 30 minutes in between^7^^-^ 11, although there is no justification in the literature for the use of this interval. For the six-minute walk test (6MWT), which evaluates functional exercise capacity, the American Thoracic Society (ATS)^11^ recommends a one-hour interval between tests. This fact shows a considerable discrepancy between the recommendation for the 6MWT and what has been used for the ISWT.

The ISWT imposes a progressive physiological stress on the tested subject. Nonetheless, there is no consensus about the ideal rest time between two ISWTs for cardiovascular variables and perceived exertion to return to baseline values. Therefore, the aim of this study was to verify whether 30 minutes of rest between two ISWTs are enough for cardiovascular variables and perceived exertion to return to their baseline status in apparently healthy individuals in a broad age range.

## Method

### Study design and subjects

A cross-sectional study was performed from March 2009 to October 2011 with 334 apparently healthy subjects included from two convenient samples: 1) elderly (age >60) participants of a project that investigated the health conditions of the elderly in Londrina, Paraná, Brazil (EELO project, Study about Aging and Longevity); and 2) students and employees from two universities in Londrina.

The study was approved by the Research Ethics Committee (PP/00007/09) of Universidade Norte do Paraná (UNOPAR), Londrina, PR, Brazil, and all participants gave written informed consent.

The inclusion criteria were: subjects from both genders; age ≥18 years old; normal lung function; absence of serious and/or unstable diseases; absence of musculoskeletal disorders that could limit performance during the test. The participants who were unable to either understand or perform any procedure during the protocol or who requested to leave the study for any reason were excluded.

### Procedures

A questionnaire was applied to investigate general health condition, medications in use and the regularity of physical activity of the subjects. Body weight and height were measured with the Filizola^(r)^ scale (Filizola, São Paulo, Brazil). The measurements were used to calculate the body mass index (BMI).

Maximal exercise capacity was evaluated by the ISWT. The test was performed twice with 30 minutes of rest in between, a period in which subjects were instructed to remain at rest. Both tests were conducted on a straight, level 10-meter path with two cones positioned 0.5 meter from each end of the route. Participants were instructed to walk according to the speed dictated by beeps, with an initial speed of 0.5 meter per second (m·s^-1)^ and increments of 0.17 m·s^-1^ every minute. The increase in speed was always indicated by a triple beep. An adaptation was made in relation to the protocol of Singh et al.^5^, which consisted of the extrapolation of the 12 levels of speed when necessary^8^ to avoid a ceiling effect and to ensure maximal exercise, since the study included healthy individuals. Both ISWT were performed by the same physical therapist or a trained physical therapy student. The initial explanation was standardized and no phrases of encouragement were given to the participants during the test. The ISWT was stopped when the participants presented one of the following conditions: inability to maintain the required speed due to dyspnea or fatigue; failure to complete the route in the time allowed for two consecutive times. The reference values for ISWT proposed by Probst et al.^8^ were used to calculate the distance walked in percentage of predicted (% pred), and the largest distance walked was considered for the analysis.

Heart rate, arterial blood pressure, dyspnea, and perception of leg fatigue were evaluated immediately before and after both tests. Heart rate was measured using a HR monitor (Polar Electro Oy, FI-90440 KEMPELE, Finland). Subsequently, the equation used for the prediction of maximum HR was calculated according to Tanaka et al.^12^. The maximal heart rate predicted for those subjects in use of beta-blockers medication was calculated as previously described^13^. In addition, the heart rate reserve (HRR), double product (DP), and chronotropic incompetence (CI) were calculated^14^. Regarding the CI, a percentage of heart rate reserve (%HRR) below 80% was considered abnormal^14^^,^ 15.

Arterial blood pressure was evaluated using a stethoscope (Welch Allyn/Tycos, Germany) and a sphygmomanometer (Welch Allyn/Tycos, Germany). Dyspnea and perception of leg fatigue were evaluated by the Modified Borg Scale^16^ (Borg D and Borg F, respectively).

### Statistical analysis

For data analysis, subjects were allocated to 6 groups according to age: G1: 18-29 years; G2: 30-39 years; G3: 40-49 years; G4: 50-59 years; G5: 60-69 years and G6: ≥70 years. Data normality was verified using the Shapiro-Wilk test. Due to the non-normal distribution of most variables, non-parametric statistics were used. Therefore, the descriptive analysis of the data was represented by median and interquartile range [25%-75%]. The intragroup comparisons of variables in the first and second ISWT were performed using the Wilcoxon test. The characteristics of the subjects, ISWT performance, as well as the difference between the beginning of the second and the beginning of the first test (delta) of the HR, %HRR, systolic blood pressure (SBP), diastolic blood pressure (DBP), Borg D, and Borg F were compared between groups using the Kruskal-Wallis test with Dunn's post-test. The Spearman correlation coefficient was used to assess the correlation between age and the deltas of the same variables described above. For gender prevalence identification between groups, the Chi-square test was used. The statistical significance was p<0.05 for all tests. Data were analyzed using the statistical program GraphPad Prism 5.0 (GraphPad Software Inc., San Diego, CA, USA). Calculation of the power of the study was performed (GPower 3.1^(r))^, demonstrating an equal or greater value than 0.95 for all comparisons.

## Results

The total sample consisted of 334 subjects, 152 men and 182 women. The age of participants ranged from 18 to 83 years old. The spirometry demonstrated that subjects had normal lung function (forced expiratory volume in the first second (FEV_1_): 96 [88-104]%pred; forced vital capacity (FVC): 94 [85-103]% pred; FEV_1_/FVC ratio: 88 [82-93]% pred). The baseline characteristics of the studied sample are described in Table 1. Regarding comorbidities among participants, hypertension (24%), rheumatic diseases (12%), and vascular problems (11%) were the most prevalent. Other comorbidities were reported: dyslipidemia (10%), stable heart disease (10%), diabetes mellitus (10%), thyroid disorders (9%), and osteoporosis (7%). Concerning medication use, 63% of the total sample (51% were elderly) used continuous medication (36% to control blood pressure). Regarding the level of physical activity, 61% of participants (207 subjects) were not involved in regular physical activity.

**Table 1.** Baseline characteristics of the studied subjects, comorbidities and use of Beta-blockers (n=334).

Variables	Total sample	G1	G2	G3	G4	G5	G6	p value
Gender (M/F)	152/182	19/26	18/24	26/27	9/10	40/47	40/48	0.67
Age (years)	61 [39-70]	23 [21-25]	35 [321-38]	45 [42-48]^*^	54 [51-56]^*#§^	65 [62-67]^*#§¥^	74 [72-77]^*#§¥‡^	<0.0001
Height (m)	1.63 [1.56-1.70]	1.70 [1.63-1.76]	1.65 [1.60-1.73]	1.64 [1.60-1.75]	1.63 [1.55-1.72]	1.60 [1.53-1.67]^*#§^	1.58 [1.50-1.66]^*#§^	<0.0001
BMI (kg·m^-2^)	26 [23-28]	22 [21-26]	26 [22-28]	26 [24-28]^*^	28 [25-30]^*#^	26 [24-30]^*#§^	26 [24-28]^*^	<0.0001
Comorbidities n (%)	196(58)	3(7)	8(19)	18(34)	8 (42)	67(77)	69(78)	
None	138(42)	42 (93)	34(81)	35(66)	11(58)	20(23)	19(22)	
1 or 2	117(35)	3(7)	8(19)	13(24)	8(42)	53(61)	52(60)	
3 or more	79(23)	0(0)	0(0)	5(9)	0(0)	14(16)	17(19)	
Beta-blockers n(%)	60(18)	0(0)	0(0)	0(0)	0(0)	23(26)	37(42)	

Age, Height and BMI values are shown as median and interquartile range [25% - 75%]. Groups separated by age: G1: 18-29 years; G2: 30-39 years; G3: 40-49 years; G4: 50-59 years; G5: 60-69 years; G6: ≥70 years. M: male; F: female; BMI: body mass index; Comorbidities presented in number (n) and percentage (%); Beta-blockers use presented in number of individuals (n) and percentage (%);

* p<0.05 vs G1;

# p<0.05 vs G2;

§ p<0.05 vs G3;

¥ p<0.05 vs G4;

‡ p<0.05 vs G5.

Regarding HR values, the initial heart rate (HRi) of the second test was higher than the first one in the total sample (92 [83-101] bpm versus 80 [71-90] bpm, respectively; p<0.0001) and also in all groups (p<0.0001), as can be seen in Table 2. It was observed that 87% of the total sample showed higher HRi values in the second ISWT compared to the first one. A small part of the sample presented higher HRi in the first ISWT than in the second one (11%) and only 2% had the same HRi values in both tests. In fact, the HR variation (final - initial heart rate ISWT) was significantly higher in the first ISWT compared to the second one in the total sample and G3, indicating that the majority of subjects began the second ISWT with higher values of HR (Table 2).

**Table 2.** Cardiovascular variables and perceived exertion between the two ISWTs.

Variables	ISWT1						
	Total Sample	G1	G2	G3	G4	G5	G6
HRi	80[72-90]	92[84-98]	87[78-97]	84[76-92]	74[68-88]	76[80-86]	75[69-86]
SBPi	120[110-130]	120[110-120]	110[110-120]	120[110-130]	120[120-130]	130[120-140]	130[120-140]
DBPi	80[70-80]	80[70-80]	70[70-80]	80[70-80]	80[80-80]	80[80-90]	80[70-80]
HRR	76[52-94]	96[83-109]	93[83-105]	91[80-98]	82[60-98]	63[51-82]	51[38-61]
%HRR	93[71-108]	94[83-105]	99[86-106]	98[89-108]	80[66-99]	81[66-122]	84[61-121]
CI n(%)	118(35)	13(28)	10(23)	4(9)	8(42)	41(47)	42(47)
DP (bpm x mmHg)	9960[8650-11430]	10560[9525-11820]	9475[8493-11475]	9750[8380-11640]	9240[8160-10560]	10140[8610-11445]	9735[8400-11200]
D HR (f-i)	76[52-94]	96[83-109]	93[83-105]	91[80-98]	82[60-98]	63[51-82]	51[38-61]
Borg Di	0[0-0]	0[0-0,2]	0[0-0]	0[0-0]	0[0-0]	0[0-0]	0[0-0]
Borg Fi	0[0-0]	0[0-0,3]	0[0-0]	0[0-0]	0[0-0]	0[0-0]	0[0-0]
**Variables**	**ISWT2**						
	**Total Sample**	**G1**	**G2**	**G3**	**G4**	**G5**	**G6**
HRi	92[83-101]^*^	97[88-109]^*^	98[89-111]^*^	96[88-109]^*^	90[83-102]^*^	90[80-98]^*^	85[75-94]^*^
SBPi	120[110-130]	110[110-125]	115[108-120]	120[110-120]	120[110-130]	130[120-140]	120[110-140]
DBPi	80[70-80]	80[70-80]	70[70-80]	80[70-80]	80[80-90]	80[80-90]	80[70-80]
HRR	70[52-87]^*^	89[81-104]	86[73-93]	81[71-91]^*^	76[43-93]	64[46-72]	50[34-66]
%HRR	101[82-117]^*^	99[92-106]	104[94-114]	105[92-115]	90[72-112]	95[77-141]	101[61-145]
CI n(%)	76(23)	3(7)	5(12)	6(11)	5(26)	25(28)	32(36)
DP (bpm x mmHg)	11045[9680-12740]^*^	11110[9650-12960]	10790[9575-13165]^*^	11220[9840-13050]^*^	10920[9960-12880]^*^	11570[10110-12670]^*^	10210[9240-12410]^*^
D HR (f-i)	70[52-86]^*^	89[81-104]	86[73-93]	81[91-71]^*^	76[43-93]	64[46-72]	50[34-65]
Borg Di	0[0-0]	0[0-1]	0[0-0]	0[0-0]	0[0-0]	0[0-0]	0[0-0]
Borg Fi	0[0-0]	0[0-2]^*^	0[0-0,5]	0[0-0]	0[0-0]	0[0-0]	0[0-0]

Data is shown as median [interquartile range 25% - 75%]. Groups separated by age: G1: 18-29 years; G2: 30-39 years; G3: 40-49 years; G4: 50-59 years; G5: 60-69 years; G6: ≥70 years; ISWT1: First Incremental Shuttle Walking Test; ISWT2: Second Incremental Shuttle Walking Test; HRi: Initial Heart Rate; HRR: Heart Rate Reserve; %HRR: percentage of Heart Rate Reserve; CI: Chronotropic Incompetence; DP: Double Product; Δ HR (f-i): variation between the final Heart Rate - initial Heart Rate in the ISWT; SBPi: Initial Systolic Blood Pressure; DBPi: Initial Diastolic Blood Pressure; Borg Di: Initial Dyspnea Borg; Borg Fi: Initial Fatigue Borg.

* p<0.05 vs ISWT1 (intragroup comparison).

In the analysis of initial systolic blood pressure (SBPi), no difference was observed in the total sample or in the groups (Table 2). Regarding the total sample, 33% had lower SBPi values in the second ISWT compared to the first one, 27% of participants showed higher SBPi values in the second ISWT compared to the first one, and 40% had exactly the same SBPi values in both tests. No difference was observed in relation to initial diastolic blood pressure (DBPi) (p>0.05 for all comparisons).

Regarding perceived exertion, no difference was found when comparing dyspnea sensation at the beginning of the first and second ISWT in the total sample and in all groups (p>0.05 for all comparisons), but when comparing leg fatigue sensation can be observed difference in the G1 (Table 2).

Concerning the HRR, significant lower values were found in the second test in comparison with the first test in the total sample and G3 (Table 2). The total sample presented %HRR values significantly higher in the second ISWT in comparison to the first one (Table 2). Finally, the incidence of chronotropic incompetence (%HRR below 80%) was more observed in the first ISWT than in the second ISWT, however this difference was not statistically significant (p=0.49).

Regarding the DP, the values differed significantly between the first and second test in the total sample and in G2, G3, G4, G5, and G6. Higher values were observed in these groups in the second test in comparison with the first test, as can be seen in Table 2.

The variation analysis (difference between values from the beginning of the second ISWT - values from the beginning of the first ISWT) is presented in Table 3.

**Table:** Table 3.Variation (Δ) in heart rate, blood pressure and perceived exertion between the ISWT.

Variables	Total Sample	G1	G2	G3	G4	G5	G6	p value
D%HRR	8[-1–19]	2[-2–10]	6[1–12]	3[-4–11]	9[1–16]	13[2–29]^§#^	12[-1–37]^§#^	0.0003
DHRi	10 [4–17]	8.5 [-1–20]	9 [4–22]	12 [7–22]	15 [7–21]	10 [4–17]	7 [1–13]^#*^	0.0083
DSBPi	0 [-10–10]	0 [-10–0]	0 [-10–10]	0 [-10–0]	0 [-10–10]	0 [-10–10]	0 [-20–10]	0.8512
DDBPi	0 [-10–10]	0 [-10–3]	0 [0–10]	0 [-5–0]	0 [0–0]	0 [-10–10]	0 [-10–10]	0.3828
DBorg Di	0 [0–0]	0 [0–0.3]	0 [0–0]	0 [0–0]	0 [0–0]	0 [0–0]	0 [0–0]	0.2113
DBorg Fi	0 [0–0]	0 [0–0.3]	0 [0–0]	0 [0–0]	0 [0–0]	0 [0–0]^§^	0 [0–0]^§^	0.0011

Data is shown as median [interquartile range 25% - 75%]. Groups separated by age: G1: 18-29 years; G2: 30-39 years; G3: 40-49 years; G4: 50-59 years; G5: 60-69 years; G6: ≥70 years; Δ: variation between values from the beginning of the second ISWT - values from the beginning of the first ISWT; %HRR: percentage of Heart Rate Reserve; HRi: Initial Heart Rate; SBPi: Initial Systolic Blood Pressure; DBPi: Initial Diastolic Blood Pressure; Borg Di: Initial Dyspnea Borg; Borg Fi: Initial Fatigue Borg. §p<0.05 vs G1;

# p<0.05 vs G3;

* p<.05 vs G4.

There was a significant difference in the HRi variation between G6 compared to G3 and G4 (Table 3). The %HRR variation was also different in G5 and G6 when compared to G1 and G3 (Table 3). The variation in SBPi, DBPi, and initial Borg dyspnea (Borg Di) did not show difference among the groups, but the initial Borg fatigue (Borg Fi) showed significant difference in G1 when compared with G5 and G6 (Table 3).

**Figure 1.**
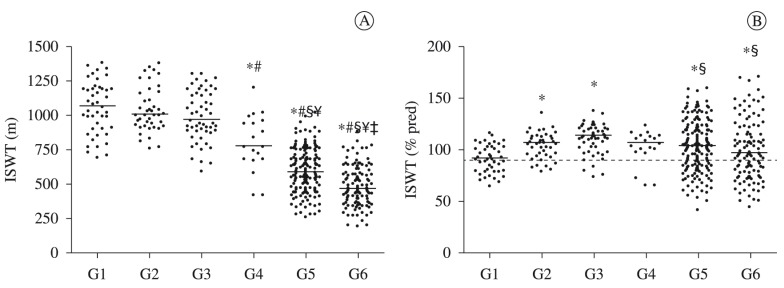
A) Incremental Shuttle Walking Test (ISWT) in all groups (G1-G6) presented in meters (test with the largest distance walked); B) ISWT in all groups (G1-G6) presented in percentage of predicted; G1: 18-29 years; G2: 30-39 years; G3: 40-49 years; G4: 50-59 years; G5: 60-69 years; G6: ≥70 years;* p<0.05 vs G1; # p<0.05 vs G2; § p<0.05 vs G3; ¥ p<0.05 vs G4; ‡ p<0.05 vs G5; The dotted line (B) represents 90% of the predicted value for the ISWT.

All groups had a good performance in the ISWT (median >90% of the predicted distance), considering the greatest walked distance, as can be seen in Figure 1. Comparing the performance in the first and second tests, the majority of the total sample (58%) demonstrated a better performance in the second ISWT. However, approximately one third of the sample showed better performance in the first test (34%), while a minority walked the same distance in both tests (8%).

Age was weak and negatively correlated only with the variation in HRi (r=-0.13, p=0.004) and Borg Fi (r=-0.21, p=0.0001) in the total sample. There were no significant correlations of age with SBPi, DBPi or Borg Di (p>0.05 for all).

## Discussion

The present study demonstrated that, regardless of age, 30 minutes of rest between two ISWTs are not enough for cardiovascular variables to return to baseline values, since most participants presented higher HR before the second test when compared to the first one (87% of the study sample). It has been previously demonstrated that parasympathetic activity, acting on HR modulation, tends to recover more slowly after intense and moderate exercise than after low-intensity exercise^17^. In addition, the difference between the HRi of the first ISWT in comparison with the second ISWT was greater in G3 and G4 than in G6. These findings agree with the literature since, according to Ogawa et al.^18^, the elderly demonstrate lower values of HR, systolic volume (SV), and cardiac output (CO) during endurance exercise than younger subjects. This is because heart rate variability (HRV) is lower in middle-aged and elderly subjects compared to younger people, both at rest and during exercise, as a result of the deleterious effects of aging on cardiac autonomic function^19^^-^ 21. Additionally, a portion of the elderly of the present study sample used beta-blocker medication, which contributes to the decrease in HR. Furthermore, these subjects presented a slightly lower performance in the ISWT than younger subjects.

Regarding other cardiac variables, the double product was significantly higher at the beginning of the second ISWT in comparison with the first one, demonstrating that 30 minutes of rest were not enough for cardiac variables to recover from exercise. Double product (SBP (mmHg) x HR (bpm)) correlates with myocardial oxygen consumption (MVO_2_), thus it is considered the most reliable indicator of heart work during continuous aerobic physical exertion^22^.

Chronotropic incompetence (%HRR below 80%), defined as the inability of the heart to increase its rate commensurate with increased activity or demand, produces exercise intolerance which impairs quality-of-life, and is an independent predictor of major adverse cardiovascular events and overall mortality^23^^-^ 25. In the present study, there was an increasing incidence of CI in the groups, according to age. These results corroborate the literature, which shows that CI is more prevalent in elderly^26^^,^ 27. The use of beta-blockers may have influenced our results, since part of the study sample used this kind of medication. However, in these cases, the correction of HR was applied to calculate the CI, as recommended in the literature^28^^,^ 29. The variation between final and initial HR in the first and second ISWT presented significant difference only in the total sample and G3. It means that a greater variation in the first test shows a greater cardiac work in the first ISWT. Another important point regarding HR is that the number of individuals in each group can justify the absence of this result in most subgroups; consequently, when the total sample was analyzed this finding may have been potentiated.

Concerning arterial blood pressure, no difference was observed in the comparison of SBPi between the two tests, as well as between the deltas. This lack of difference in the SBPi can be explained by the occurrence of some physiological manifestations of exercise that are developed late after exertion, for example, it is possible to observe an increased vasodilator reactivity up to one hour after the interruption of a maximal exercise test on a treadmill^30^. According to the literature, the SBP should increase since the beginning of exercise because of increased cardiac demand^30^. At the end of the work, the SBP should decrease according to the subsequent metabolism of substances that were released into circulation by cardiac excitatory nerves that caused the elevation of SBP^31^.

It is known that the DBP behaves differently than SBP in response to exercise with intensity that increases continuously. The DBP may have a slight oscillation between 5 and 10 mmHg of the basal value^32^^,^ 33. While the SBP increases with the increase in cardiac output, muscle arteriolar vasodilatation helps to reduce the diastolic pressure, which tends to be restored post-stress to baseline values. This is corroborated by the findings of this study, with no difference observed between the two tests, in the analysis of the total sample, the groups and the deltas.

Regarding the symptomatic response to exercise, Jones and Killian^34^ reported that the perceived exertion increases in exponential function, both in relation to the power applied and the duration of the exercise, being recovered after physical exertion. These data are similar to those found in the present study, in which subjects undergoing the exercise test showed an increase in symptoms. There was no difference in the dyspnea sensation between the two tests, but it was observed difference in leg fatigue in G1 and G5. Comparing the deltas, there was difference in fatigue (Borg Fi) between G1 and G5 and G1 and G6. The literature demonstrates that, regarding healthy individuals, perceived exertion and respiratory distress in maximal exercise increase with advancing age^35^.

With respect to the performance, it is possible to see that in the present study all groups performed maximum exercise in the ISWT, since the median of walked distance was above 90% predicted. In other words, all groups presented a value of percentage of predicted greater than 90%, which can be considered normal for healthy subjects. Moreover, the percentage of maximum HR was above 85% predicted and the percentage of HR reserve was above 80%. These findings indicate that, in fact, all groups reached maximum exercise in the test.

We believe that the gender difference between the groups did not affect the results due to the fact that the proportion of men and women remained similar in all groups. In relation to BMI, there was a difference between the younger and older groups. Despite being established in the literature that BMI can be influenced by genetic and environmental factors^36^^,^ 37, advancing age is the factor that contributes the most to the increase in body weight and consequently in BMI^37^.

We observed that 39% of subjects were physically active and most (61%) did not practice any regular physical activity. However, we believe that this fact did not affect the results of this study, because the behavior of physiological and symptomatic variables was compared before and after individually and, therefore, without interference of participant fitness.

It is important to state that a limitation of the present study was the fact that the values of SBP and DBP were recorded with 10mmHg intervals, which it may have impaired the evaluation of this variable.

As reported in the literature^9^^,^ 11 ^,^ 38 ^-^ 40, most of the sample of the present study (58%) also showed the best performance in the second ISWT, covering a larger distance in the second test. It is important to notice that the majority of subjects performed better in the second ISWT, even with higher HR values before starting the second test. This shows that 30 minutes of rest between the first and second tests seem to be enough to elicit a good performance. On the other hand, 34% showed better performance in the first test and 8% had exactly the same performance in both tests. These data and the response of some cardiovascular variables and perceived exertion contribute to the hypothesis that if a greater rest period between the two tests was given, the performance of subjects could have been even better. There is still no scientific evidence regarding the ideal rest time between two ISWTs. Thus, future research is needed, e.g. a study investigating the ideal rest time between two ISWTs for symptomatic and physiological variables to return to baseline values, enhancing the performance in the second test.

It is important to mention that the ISWT has its greatest applicability described in patients with COPD^6^. However, the ISWT has been highly used in clinical practice, as well as in scientific research, especially when there is no equipment available to assess exercise capacity in healthy subjects using the CPET. Thus, the present study makes a significant contribution to the literature regarding the use of the ISWT in healthy subjects and underscores the importance of attention to physiological variables during exercise tests in this population in order to achieve the best performance in this maximal exercise test. Moreover, future studies should explore the suitable rest time between two ISWTs in ill patients, such as patients with COPD, who can be strongly benefited.

The study allows us to conclude that 30 minutes of rest between two ISWTs are not enough for all cardiovascular variables and perceived exertion to return to baseline values in apparently healthy subjects in a broad age range. However, this period appears to be sufficient for blood pressure and performance to recover in most subjects.
